# Predictive models for delay in medical decision-making among older patients with acute ischemic stroke: a comparative study using logistic regression analysis and lightGBM algorithm

**DOI:** 10.1186/s12889-024-18855-6

**Published:** 2024-05-27

**Authors:** Zhenwen Sheng, Jinke Kuang, Li Yang, Guiyun Wang, Cuihong Gu, Yanxia Qi, Ruowei Wang, Yuehua Han, Jiaojiao Li, Xia Wang

**Affiliations:** 1 Shandong Xiehe University, Jinan City, Shandong Province China; 2 Qingdao University, Qingdao City, Shandong Province China; 3 Qilu Hospital of Shandong University, Jinan City, Shandong Province China

**Keywords:** Older patients, Stroke, Acute ischemic stroke, Medical decision-making delay, Logistic regression analysis, LightGBM algorithm

## Abstract

**Objective:**

To explore the factors affecting delayed medical decision-making in older patients with acute ischemic stroke (AIS) using logistic regression analysis and the Light Gradient Boosting Machine (LightGBM) algorithm, and compare the two predictive models.

**Methods:**

A cross-sectional study was conducted among 309 older patients aged ≥ 60 who underwent AIS. Demographic characteristics, stroke onset characteristics, previous stroke knowledge level, health literacy, and social network were recorded. These data were separately inputted into logistic regression analysis and the LightGBM algorithm to build the predictive models for delay in medical decision-making among older patients with AIS. Five parameters of Accuracy, Recall, F1 Score, AUC and Precision were compared between the two models.

**Results:**

The medical decision-making delay rate in older patients with AIS was 74.76%. The factors affecting medical decision-making delay, identified through logistic regression and LightGBM algorithm, were as follows: stroke severity, stroke recognition, previous stroke knowledge, health literacy, social network (common factors), mode of onset (logistic regression model only), and reaction from others (LightGBM algorithm only). The LightGBM model demonstrated the more superior performance, achieving the higher AUC of 0.909.

**Conclusions:**

This study used advanced LightGBM algorithm to enable early identification of delay in medical decision-making groups in the older patients with AIS. The identified influencing factors can provide critical insights for the development of early prevention and intervention strategies to reduce delay in medical decisions-making among older patients with AIS and promote patients’ health. The LightGBM algorithm is the optimal model for predicting the delay in medical decision-making among older patients with AIS.

## Background

An aging society has become a common concern in both developed and developing countries. During the last 200 years, the average human life expectancy has doubled in most developed countries. It is predicted that nearly one-fifth of the population in the world will be aged ≥ 65 by 2030 [1–3], leading to an increase in the number of patients with chronic diseases [4–6]. Stroke is considered as one of the most important chronic diseases as it is the second largest cause of death and the third largest cause of disability worldwide [7]. In 2019, the global incidence, prevalence, and mortality of stroke was 12 million, 101 million, and 7 million, respectively [8, 9]. The disease burden caused by stroke can also lead to significant losses in productivity [10], and the negative consequences of stroke are expected to increase in further years [10]. Due to the aging population, a majority of stroke patients in China are older; thus, the burden of stroke predominantly impacts this age group [11].

Acute ischemic stroke (AIS) comprises 60–70% of the total number of stroke patients [12] and has various causes, AIS can lead to blood supply disorders in the brain tissue, which can result in ischaemic necrosis, hypoxic necrosis, and brain dysfunction. The most recommended and effective treatment approved for AIS is a thrombolytic agent with recombinant tissue plasminogen activator (rt-PA) [13]. Intravenous thrombolysis with rt-PA can effectively restore cerebral tissue blood supply, rescue corresponding neurological function, and improve prognosis in patients with AIS. However, this treatment measure must be used within the optimal treatment time window of 3–4.5 h after the onset of stroke symptoms. Rt-PA treatment outside the treatment time window has a reduced thrombolytic effect and can lead to haemorrhagic transformation, thereby causing additional damage to the brain [12]. Therefore, early medical care is crucial in patients with AIS.

However, older patients with AIS do not always follow the best treatment plan [14] and often face delays in medical decision-making. This delay is characterised as a duration of more 1 h between the onset of discomfort symptoms to the patient’s initial decision to seek medical help [15, 16]. This delay represents over 50% of the total time delay and is a stage that has not yet seen effective improvement. Understanding the risks of delayed medical decision-making in older patients with AIS and performing primary prevention are critically important. However, research on factors influencing delayed medical decision-making in older patients are still lacking. In existing studies, there is still controversy over whether the level of stroke knowledge has an impact on delayed medical decision-making in patients with AIS [17, 18]. Researchers consider health literacy as an important prerequisite for health, healthy behavior, and health-related decision-making [19], however, the predictive role of health literacy on delayed medical decision-making in older patients with AIS is not yet clear. In addition, social networks are conceptualized as the group of close social relationships one has, serving as sources of advice, help, support, and companionship [20], these networks gain particular importance in older age [21]. Social support resources carried by social network have an impact on cognitive function, happiness, health management behavior and mortality of older people [22–24]. Despite this, few studies have examined the impact of social network factors delayed medical decision-making in older patients with AIS. Therefore, it is urgent to verify the impact of these variables on delay in medical decision-making among older patients with AIS; this information can aid in developing more effective methods for identifying older patients at risk of delay in medical decision-making.

Logistic regression is a statistical method used to establish predictive models and is primarily used to solve binary classification problems. It is based on the concept of a linear regression model that maps the output of the linear regression to an S-shaped curve for probability estimation and classification prediction. In practical applications, logistic regression is commonly used in fields such as marketing, medical research, and risk assessment to predict the probability of an event occurring or to make classification predictions. The LightGBM is a machine learning algorithm based on a gradient-lifting tree developed by Microsoft Research Asia. It combines the advantages of extreme gradient boosting (XGBoost) and Gradient Boosting. Notably, it shows high efficiency and accuracy when processing large-scale datasets. This alleviates the limitations of decision tree algorithms and is considered superior to machine tree algorithms, thus becoming increasingly popular among scholars for developing risk prediction models. To data, the performance of logistic regression and lightGBM algorithm in predicting risk factors for delay in medical decision-making among older patients with AIS remains still unclear.

This study aimed to establish and evaluate two mathematical models for predicting the delay in medical decision-making among high-risk older patients with AIS by analyzing demographic information, disease occurrence characteristics, stroke knowledge levels, health literacy, and social networks. To identify the high-risk factors for delayed medical decision-making among older patients with AIS in the early stages and provide a theoretical basis for specifying relevant intervention measures, reducing medical delays, and promoting patient health. Simultaneously applying LightGBM to the stroke delayed medical decision-making provides new directions for future research.

## Materials and methods

### Participants

Data were obtained between October 2021 and June 2022 from the neurology wards of four tertiary hospitals in Qingdao, China. Convenience sampling was used to assess the physical and mental health of older patients with AIS prior to stroke onset. According to the formula, $Z\alpha ?\pi ?1-\pi ?/\delta ?$ [25], the allowable error between sample rate and unknown population rate was 5%, $\alpha$ was 0.05 and$Z\alpha$was 1.96. Considering the high delay rate of medical decision-making among older patients with AIS, the rate was taken as the highest value, which was 0.85 in precious studies [26]. A total of 309 patients were included in the study.

The inclusion criteria were patients who were (1) aged over 60 years, (2) confirmed to have AIS, and (3) agreed to participate in the study. The exclusion criteria were patiens with (1) severe organ diseases (severe myocardial infarction, renal failure, hepatitis, cancer, etc.) and mental illnesses (alzheimer’s disease, senile dementia, schizophrenia, or serious mental disorders caused by physical infections, endocrine disorders, nutritional and metabolic disorders, etc.), (2) unclear consciousness and inability to communicate, (3) could not perceived symptom time and medical decision-making time (nor could their family memvers), or (4) experienced cognitive impairment symptoms during seizures, which caosued them to be unable to make decisions themselves.

### Measurements

#### Demographic characteristics

Demographic data, including age, sex, education level, and marital status, were obtained through questionnaires and confirmed by checking the patients’ medical records. To gather disease information, we collected information including data on participants’ symptoms, National Institutes of Health Stroke Scale (NIHSS) score, onset time, location, first decision to seek medical attention. Whether a patient presents a medical decision-making delay was determined based on whether the time interval between the onset of symptoms and the initial decision to seek medical exceeded 1 h [15, 16].

#### Previous stroke knowledge

The 40- question Stroke-related Knowledge Questionnaire (SKQ), created by Yao Qiping in 2016, was used to evaluate patients’ previous level of stroke knowledge [27]. The questions focused on stroke symptoms, first-aid measures, risk factors, safe medication, healthy behaviours, and knowledge of rehabilitation. Each question is assigned a score of either 0 or 1, with 1 point given for correct answers and 0 foe incorrect ones. The maximum total score is 40 and Cronbach’s α is 0.858.

#### Health literacy

The Health Literacy Management Scale (HeLMS) for chronic disease patients was rendered into the Chinese language by Sun Haolin in 2012 [28]. This scale consists of 24 items, and it reflects an individual’s health literacy status on four aspects: health information ability, health information assistance ability, health willingness, and economic support. The Likert 5-point scoring method is employed, with values ranging from ‘absolutely impossible’ (1 point) to ‘no difficulties’ (5 points). The total score ranges from 24 to 120 points. Higher scores indicated higher levels of health literacy. Cronbach’s α is 0.977.

#### Social network

The Lubben Social Network Scale (LBSNS) has been used previously to assess family, friends, and social networks among older patients with AIS [29]. It is currently the most widely used tool for assessing social isolation among older adults. The scale comprises 11 questions, each with a specific score. Lower scores indicate a higher degree of social isolation. If the total score is less than or equal to 19 points, there may be a risk of isolation. Cronbach’s α is 0.92.

### Statistical analyses

Data were analyzed using IBM SPSS. The Harman single factor test was used to test for common method bias. Econometric data that followed a normal distribution were listed as mean ± standard deviation, and the t-test was used for comparative analysis. Data not adhering to a normal distribution were described using the median and interquartile range, and comparisons and analyses are conducted using the rank sum test for two independent samples in non-parametric testing. Qualitative data were listed as frequency and percentage, and either the chi-square test or Fisher’s exact test was used foe comparison and analysis. These statistical methods were employed to investigate potential differences in each risk factor between two patient groups: those making timely decisions (≤ 1 h) and those with delayed decision-making (> 1 h). Correlation analysis was conducted and VIF values were calculated to determine collinearity between variables. A *P*-value < 0.05 obtained using logistic regression analysis was deemed statistically significant. The LightGBM algorithm analysis was performed using Pycharm 2023.3.

## Results

### Sample characteristics and clinical data

A total of 309 valid questionnaires were collected. As shown in Tables 1 and 58.6% of patients were males, and 60.2% of patients were between 60 and 70 years of age. The proportion of patients in rural and urban was equivalent; 45% were retired, 33.3% had a per capita monthly income ranging from 3000 to 5000 yuan, and only 1.3% had received higher education. Overall, most people lived with their children or spouses, and approximately half of the respondents underwent physical examination once a year.

**Table 1:** Characteristics of the study population and clinical data

Characteristic	Delay	None delay	*n* (%)	χ^2^	*P*-value	M ± SD
Gender				0.962	0.367	
Male	139	42	181 (58.6)			
Female	92	36	128 (41.4)			
Age				10.161	**0.006**	69.36 ± 6.91
60–70	149	37	186 (60.2)			
70–80	65	27	92 (39.8)			
≥ 80	17	14	31 (10.0)			
Residence						
Rural	117	35	152 (49.2)			
Urban	114	43	157 (50.8)			
Occupation				1.432	0.69	
Worker	76	23	99 (32.0)			
Non worker	26	6	32 (10.4)			
Retired	101	38	139 (45.0)			
Unemployed	28	11	39 (12.6)			
Per capita monthly income (CNY * )				9.987	**0.019**	
<1000	51	18	69 (22.3)			
1000–3000	77	12	89 (28.8)			
3000–5000	70	33	103 (33.3)			
≥ 5000	33	15	48 (15.5)			
Education				1.415	0.516	
Junior high school and below	166	51	217 (70.2)			
High school and junior college	62	26	88 (28.5)			
Bachelor degree or above	3	1	4 (1.3)			
Living						
Living with children or spouses	207	71	278 (90)	0.266	0.923	
Living Alone	21	6	27 (8.7)			
Others (sanatoriums, etc.)	3	1	4 (1.3)			
Physical examinations				10.622	**0.012**	
More than or equal to two times a year	14	1	15 (4.9)			
Once a year	101	47	148 (47.9)			
Every few years,	64	22	86 (27.8)			
Never	52	8	60 (19.4)			
Family history of stroke				0.052	0.82	
Yes	16	6				
No	215	72				
First stroke				9.457	**0.002**	
Yes	46	29	75			
No	185	49	234			
Stroke severity				25.336	**< 0.001**	
Minor (NIHSS ≤ 4)	144	23	167			
Moderate to severe (NIHSS > 4)	87	55	142			
Mode of onset				2.667	**0.008**	
Slow onset	160	66				
Acute onset	71	12				
Reactions from others				62.287	**< 0.001**	
Advised the patients to self-treat	94	0	94			
Advised the patients to seek treatment	132	75	207			
Patients did not tell anyone about their symptoms	5	3	8			
Suffer from other chronic diseases				0.743	0.389	
Yes	163	59	222			
No	68	19	87			
One person present				0.241	0.624	
Yes	193	67	260			
No	38	11	49			
Stroke recognition				39.914	**< 0.001**	
Yes	40	42	82			
No	191	36	227			
Onset location				1.915	0.430	
Home	204	71	275			
Workplaces	11	1	12			
Public places	16	6	22			
Previous stroke knowledge	/	/	/	-6.776	< 0.001	21.719 ± 7.405
Health literacy	/	/	/	-3.953	< 0.001	130(115,138)†
Social network	/	/	/	-2.110	0.036	26.580 ± 6.105

n: The number of samples in this group; χ^2^:Pearson’s chi-square test; M ± SD: Mean ± Standard Deviation; *CNY: Chinese Yuan, United States dollar/Chinese Yuan ≈ 7.25; †: Non normal distribution is described using median (interquartile range); Mode of onset-Slow onset: there is a long time between the onset of symptoms and the onset of obvious symptoms; Mode of onset-Acute onset: rapid and obvious clinical manifestations appear within a short period of time

### Delayed medical decision-making among older patients with AIS

In this study, the median delay in medical decision-making in older patients with AIS was 8 h. Of the 309 patients, 74.76% presented medical decision-making delay (> 1 h), and only 78 patients presented early decision delay (≤ 1 h). Overall, 110 (35.60%) and 137 (44.34%) patients had a decision delay of 3 h or less and between 3 and 6 h, respectively, whereas 17 patients (15.5%) had a decision delay of > 24 h.

### Univariate analysis of decision-making delay

The results of the normality test indicate that the previous knowledge level and social networks follow a normal distribution, while the level of health literacy follows a skewed distribution. There were statistically significant differences in the medical decision-making time of older patients with AIS in terms of age, per capita monthly income, physical examinations, first stroke, stroke severity, reaction from others, stroke recognition, previous stroke knowledge, health literacy, and social networks (*P* < 0.05). This is shown in Table 1.

### Multicollinearity test

**Fig. 1:**
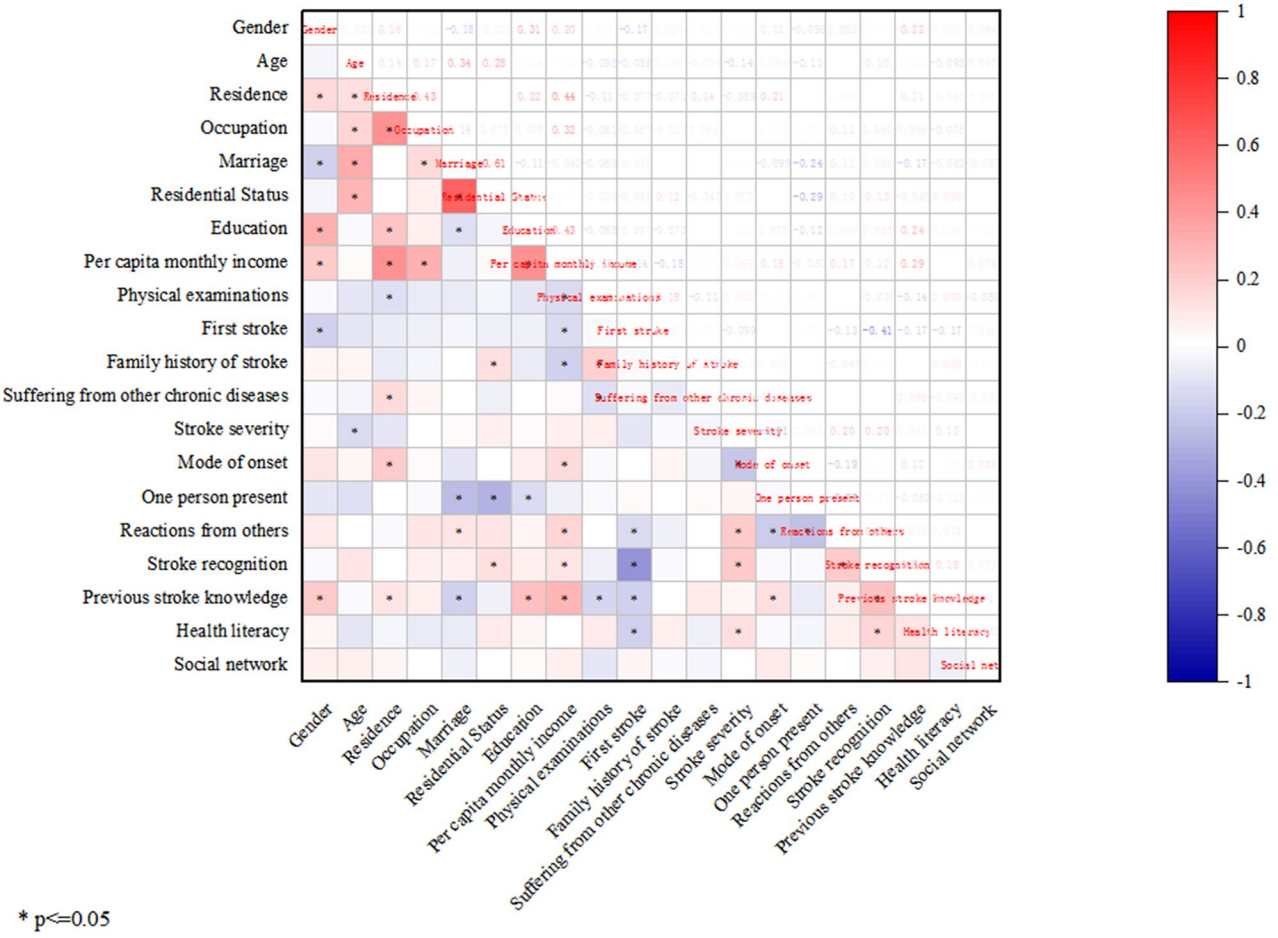
Heatmap of the correlation coefficient of variables

We conducted a correlation analysis of the variables to avoid collinearity. The correlation heat map shows the correlation between the variables and combination of statistically significant variables (Fig. 1). This indicates that a correlation exists between some of the variables; however, the degree of correlation was low (< 0.5). The variance inflation factor (VIF) was used to test variable multicollinearity. A VIF value of < 5 indicates weak multicollinearity. Table 2 shows that the VIF values of each variable were close to 1, indicating weak collinearity of the variables and, consequently, demonstrating that the selected variables could effectively prevent the negative impact of feature collinearity on the classification performance of the model.

**Table 2:** VIF test values

Variables	VIF value	Variables	VIF value	Variables	VIF value
Gender	1.271	Per capita monthly income	1.669	Mode of onset	1.184
Age	1.245	Physical examinations	1.122	Reactions from others	1.183
Residence	1.570	First stroke	1.299	Stroke recognition	1.419
Occupation	1.349	Family history of stroke	1.119	Previous stroke knowledge	1.307
Marriage	1.936	Other risk factors	1.069	Health literacy	1.124
Residential Status	1.815	Stroke severity	1.179	Social network	1.068
Education	1.552	One person present	1.181		

### Prediction model based on logistic regressive analysis

The dependent variable was whether older patients with AIS experienced delay in medical decision-making, and the independent variables were selected for inclusion in the model based on literature review and univariate analysis results. The variables underwent forward (conditional) entry regression analysis, with a criterion for variable inclusion set at 0.05 and a criterion for exclusion set at 0.10. The result of the Hosmer-Lemeshow test indicated that the *p*-value was greater than 0.05, suggesting that the current data were been adequately captured, and thus the model exhibited good fit.

The results of the binary logistic regression analysis are presented in Table 3. The possibility of delayed decision-making in patients with moderate-to-severe stroke (odds ratio [OR]: 0.301; confidence interval [CI]: 0.139–0.653), sudden-onset stroke (OR: 2.648; CI: 1.034–6.779), or recognition of symptoms as stroke symptoms by patients or their families was lower (OR: 0.275; CI: 0.123–0.616) compared to other patients. Moreover, patients with higher levels of stroke knowledge (OR: 0.882; CI: 0.832–0.935) and health literacy (OR: 0.972; CI: 0.950–0.995), as well as denser social networks (OR: 0.899; CI: 0.842–0.961), were less likely to make delayed medical decisions compared to other patients.

**Table 3:** Logistic regressive analysis in older patients with AIS

Variable	Β	SE	Wald χ^2^	*P*	OR (95% CI)
Stroke severity	-1.199	0.395	9.227	0.002	0.301 (0.139, 0.653)
Mode of onset	0.974	0.480	4.123	0.042	2.648 (1.034, 6.779
Stroke recognition	-1.292	0.412	9.834	0.002	0.275 (0.123, 0.616)
Previous stroke knowledge	-0.126	0.030	18.093	0	0.882 (0.832, 0.935)
Health literacy	-0.029	0.012	5.98	0.016	0.972 (0.950, 0.995)
Social network	-0.106	0.034	9.964	0.002	0.899 (0.842, 0.961)

B, beta; SE, Standard error; OR, odds ratio; and CI, confidence interval

### Prediction model based on the LightGBM algorithm

**Fig. 2:**
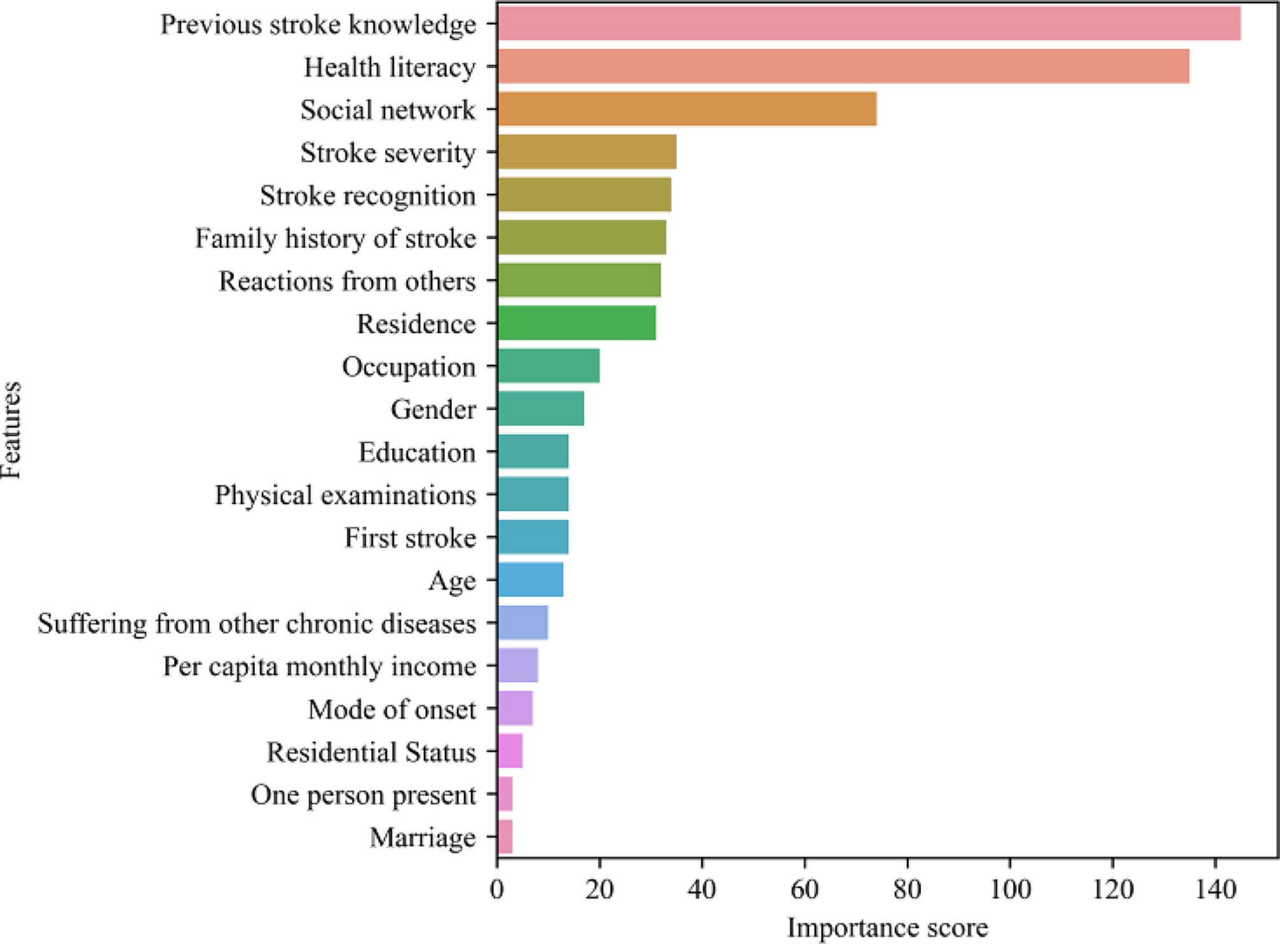
Importance scores of features presented by LightGBM model

To identify key parameters influencing delayed medical decision-making in older patients with AIS, the importance scores of features in the LightGBM model were generated. Previous stroke knowledge, health literacy, social networks, stroke recognition, reactions from others, and stroke severity were the most important parameters for predicting the delay in medical decision-making. (Fig. 2).

### Evaluation of the prediction performance of the two models

The performance of the two prediction models was evaluated using five parameters including Accuracy, Recall, F1 Score, Area Under Curve (AUC) and Precision. The results are shown in Table 4. The LightGBM model demonstrated superior performance, achieving the higher AUC of 0.909. Given that the LightGBM model exhibited the ideal performance across the two algorithms, it was deemed the best model.

**Table 4:** The result of comparison between two prediction models

Model	Accuracy	Recall	F1 Score	AUC	Precision
Logistic regressive model	0.806	0.955	0.875	0.848	0.808
LightGBM model	0.806	0.977	0.878	0.909	0.796

## Discussion

Our study included 309 participants aged ≥ 60 years and the results showed that approximately three-quarters of the patients experienced delayed medical decisions-making. Logistic regression analysis and LigitGBM algorithm were used to establish predictive models and screen for stroke-related risk factors. The two predictive models jointly reported that disease severity, stroke recognition, previous stroke knowledge, health literacy, and social networks were factors influencing delayed medical decision-making in older patients with AIS. In the logistic regression model, ‘stroke onset characteristics’ was statistically significant in determining whether there was a delay in medical decision-making. In the GBM algorithm, the ‘response of others’ was of high importance. We also evaluated the performance of both models and found that GBM algorithm outperformed logistic regression.

Research reports have shown that the delay rate in medical decision-making ranges from 40.9 to 71.5%. A study conducted in Malaysia showed that the delay rate in medical decision-making for patients with AIS in hospitals was 54.9% [15]. Zhang et al. investigated the delay status of patients with AIS in rural areas and found that the delay rate for this group of patients was 71.5% [16]. However, in this study, the delay rate for medical decision-making in older patients with AIS was 74.76%, which is high. The median decision delay (480 min) in seeking treatment for older patients with AIS in this study is much higher than previous research results [15–17, 30, 31]. The main reason is the difference in the study population, which means that the delay in medical decision-making among older AIS patients is more severe than the median delay in decision-making among AIS patients in adult studies [15, 17, 30, 31] or in rural areas [16]. Older patients are less sensitive to physical symptoms and have higher pain thresholds, and most older patients fear hospitalization and are unwilling to inconvenience their families, leading to delays in seeking medical care [32]. Additionally, longitudinal research reports indicate that with increasing age, older adults experience declines in financial skills [33], health literacy [34], and medical decision-making abilities [35, 36], leading them to exhibit a greater tendency for decision delay compared to younger adults [37]. However, the physical function of the older gradually declines with age [38], and the damage caused by delayed medical treatment is more serious; therefore, it is necessary to manage and regulate the intervenable factors that affect medical decision-making delays to reduce treatment delays for older patients who have undergone a stroke.

Our results show that stroke severity and stroke recognition are factors influencing the delay in medical decision-making among older patients with AIS, which is consistent with the findings of Lim et al. [15]. Mild symptoms, such as mild dizziness and headaches, confuse older people with their pre-existing symptoms of other common chronic diseases, including high blood pressure. Older people may rest, take medication, and observe themselves at home before going to the hosipital, leading to delayed decision-making in seeking medical attention. In contrast, if the symptoms are severe or older patients perceive them as having a stroke, they may seek medical attention promptly [17]. As bodily sensations beyond the ‘normal’ range may trigger a self-evaluation process, unfamiliar or severe symptoms can attract the patient’s attention and lead to timely seeking of professional medical assistance [39]. Ivynian et al. [39] believed that the inability to correctly recognize stroke symptoms indicated cognitive dissonance, which may lead to emotional responses of avoidance and denial.

The level of previous stroke knowledge was an important factor affecting delayed medical decision-making in older patients with AIS, ranking first in importance in the decision tree model. However, studies have suggested no correlation between high levels of stroke knowledge and timely decision-making [18, 40, 41], possibly because they only use a single dimension to evaluate stroke knowledge levels [17]. However, in our study, patients’ knowledge was evaluated by the symptoms of stroke, first-aid measures, risk factors, safe medication, healthy behaviour, and rehabilitation knowledge. Higher knowledge scores indicated that older patients were better at recognizing stroke abilities and emergency awareness, ensuring that they could make timely medical decisions when symptoms appear. Moreover, research has found that the positive effect of publishing stroke knowledge through public health campaigns has given unexpected results. Therefore, more in-depth research is needed to formulate more sustainable, multilevel, practicable, and effective health education strategies for older patients with AIS [15]. Additionally, broad, multi-levelled, practicable, and effective health education strategies, such as geriatric health related lectures and simulation, are required instead of focusing too narrowly on stroke symptoms alone [42]. These strategies are warranted to ensure that improved knowledge can be translated into symptom recognition and correct seeking behaviour when symptoms appear [18].

This study found a negative correlation between the health literacy level of older patients with AIS and delays in medical decision-making. Health literacy is a complex concept that involves many aspects of individual skills [43]. Patients with high levels of health literacy demonstrate rich motivation, ability, cognition, and social skills to acquire, understand, and apply information to promote and maintain good health [44, 45], actively manage their own health, and making timely medical decisions. Therefore, improving the health literacy level of the older population at high-risk for stroke can be an effective strategy to reduce delays in medical decision-making for stroke patients. Community workers should use scientific tools to assess the health literacy levels of older high-risk stroke populations and develop personalized intervention measures based on patient characteristics and health literacy levels to improve individual health literacy levels and reduce delays in medical decision-making.

Previous studies have focused on the positive effects of social support on timely medical treatment [16, 46–48]. One Chinese study demonstrated that social support was the only feature in the prediction model that affected decision-making delays in rural stroke patients [16], which is consistent with our studies. A correlation exists between social networks and delayed medical decision-making in older patients with AIS. Gao constructed a simulation model and found that the effective scale of social networks was the strongest indicator affecting the number of prehospital delays [24]. Older patients with large, effective social networks can access diverse health information and support resources. Others in the social network can participate in health activities with patients and help the older patient identify their symptoms and take urgent action more quickly. Moreover, socialising can maintain the activity-related performance of the older brain and improve emergency response speed. Therefore, expanding the social network of the older population at high-risk for stroke can help to reduce delay in medical decision-making. Community health organisations should encourage this population to actively participate in social activities, regularly perform physical exercise, engage in activities that involve communication. This can help create a positive social support environment, and establish stable social networks for older adults.

Our study had several limitations. Firstly, cross-sectional studies cannot determine causal relationships. Longitudinal or intervention studies should be conducted to further determine the influencing factors of delayed medical decision-making in older patients with AIS. Secondly, the main variables were dependent on the description provided by patients, which may result in biases related to social expectations and reporting. Lastly, there may be potential mutual influences between variables, and further in-depth research on the underlying mechanisms of influencing factors is needed in the future.

## Conclusion

In this study, logistic regression analysis and the LightGBM algorithm were applied to screen the relevant risk factors in older patients with AIS and establish prediction models. We discovered that stroke severity, stroke recognition, stroke onset characteristics, response of others, previous stroke knowledge, health literacy, and social networks were factors impacting delayed decision-making in older patients with AIS. This finding demonstrates the need for targeted prevention interventions that improve the stroke knowledge and health literacy among the older patients. It also emphasizes the need for expanding social networks to reduce the occurrence of delayed medical decision-making for older patients with AIS, with potential implications for future public health efforts. And the prediction effect of the LightGBM algorithm for Delayed medical decision-making among older patients with AIS was more accurate than that of logistic regressive analysis.

## Data Availability

The datasets used and/or analysed in the current study are available from the corresponding author upon reasonable request.

## Abbreviations

AIS: Acute ischemic stroke
LightGBM: Light Gradient Boosting Machine algorithm
rt-PA: Recombinant tissue plasminogen activator
VIF: Variance inflation factor
